# Therapeutic Mechanism and Key Active Ingredients of Shenfu Injection in Sepsis: A Network Pharmacology and Molecular Docking Approach

**DOI:** 10.1155/2022/9686149

**Published:** 2022-08-26

**Authors:** Huajing Yuan, Yang Liu, Kai Huang, Hao Hao, Yi-tao Xue

**Affiliations:** ^1^Shandong University of Traditional Chinese Medicine, Jinan 250014, China; ^2^Affiliated Hospital of Shandong University of Traditional Chinese Medicine, Jinan 250014, China

## Abstract

At present, although the early treatment of sepsis is advocated, the treatment effect of sepsis is unsatisfactory, and the mortality rate remains high. Shenfu injection (SFI) has been used to treat sepsis with good clinical efficacy. Based on network pharmacology, this study adopted a new research strategy to identify the potential therapeutic targets and key active ingredients of SFI for sepsis from the perspective of the pathophysiology of sepsis. This analysis identified 28 active ingredients of SFI based on UHPLC-QQQ MS, including 18 ginsenosides and 10 aconite alkaloids. 59 targets were associated with the glycocalyx and sepsis pathways. Based on the number of targets related to the pathophysiological process of sepsis, we identified songorine, ginsenoside Rf, ginsenoside Re, and karacoline as the key active ingredients of SFI for the treatment of sepsis. According to the cluster analysis of MCODE and the validation on the GEO dataset, LGALS3, BCHE, AKT1, and IL2 were identified as the core targets. This study further explored the therapeutic mechanism and the key active ingredients of SFI in sepsis and provided candidate compounds for drug development.

## 1. Introduction

Sepsis is a series of organ dysfunction syndromes caused by dysregulated infection response. In addition to systemic inflammatory response and infection of the primary lesion, severe patients may also experience shock and multiple organ failure [1]. Although we advocate early and timely treatment for sepsis patients, the mortality rate remains high. Global epidemiological studies have shown that 29.5% of ICU patients had sepsis during their hospitalization. The ICU mortality rate of sepsis patients was 25.8%, which was significantly higher than that of general ICU patients (16.2%) [2]. It can be seen that sepsis is still a problem in modern medicine.

Shenfu injection (SFI), derived from the traditional Chinese medicine (TCM) Shenfu decoction, is extracted from *Talinum paniculatum (Jacq.)* (hongshen) and *Aconitum carmichaeli Debx* (fuzi). In clinical treatment, SFI has been widely used to treat a variety of diseases including sepsis [3, 4], shock [5], and heart failure [6] with good curative effects. Experimental evidence supports that SFI is effective in improving immune function in patients with sepsis [7, 8], reducing septic lung injury [9], and improving the prognosis for patients with sepsis [7, 10, 11]. In addition, SFI can reduce the release of inflammatory factors such as TNF-*α*, reduce the inflammatory response, and inhibit apoptosis by downregulating the MEK and ERK signaling pathways [12]. Preliminary experiments and clinical research of our research group have shown that SFI improves pulmonary vascular permeability [13] in septic rats, inhibits the degradation of the glycocalyx, reduces fluid retention in septic shock patients [14], and improves tissue perfusion in early volume resuscitation [15].

**Figure 1:**
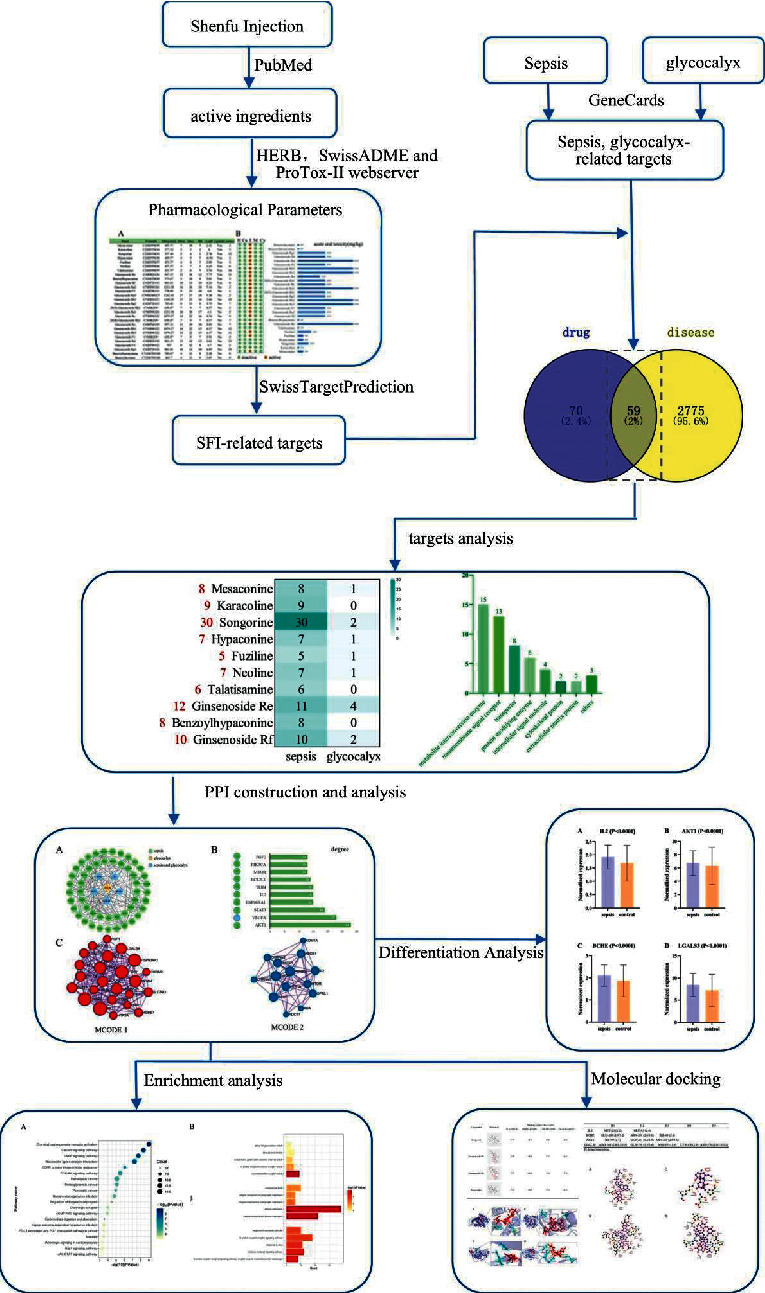
Flow chart of this study.

Given the multi-ingredient, multitarget, and multiway characteristics of TCM, we use network pharmacology to describe its mechanism of action. Network pharmacology combines various disciplines including traditional pharmacology, systems biology, and computer technology [16]. Studies have confirmed that in-silico technology can effectively explore and determine the interaction mechanism between molecules and proteins, and between proteins [17–19]. A variety of in-silico techniques, including molecular docking, can explore the molecular mechanism of disease [20], therapeutic mechanism of drugs for diseases [21, 22], drug resistance mechanisms [23], and other mechanisms of action. Therefore, we used molecular docking to imitate the interaction of drug-effective molecules with disease targets. Due to glycocalyx being the key mechanism of sepsis [24], we started from the pathophysiological mechanism of sepsis and explored the therapeutic mechanism of SFI involved in glycocalyx and sepsis based on network pharmacology and molecular docking. The workflow of this study is shown in Figure 1.

## 2. Materials and Methods

### 2.1. Determination of the Main Active Ingredients of SFI and Evaluation of Pharmacological Parameters

The PubMed database (http://www.ncbi.nlm.nih.gov/pubmed) was used to retrieve the main active ingredients of SFI in plasma. Through a search of the PubMed database, we found the latest research on UHPLC-QQQ MS (ultra-high performance liquid chromatography-triple quadrupole tandem mass spectrometry) for SFI [25]. Based on this UHPLC-QQQ MS research, we obtained 28 active ingredients of SFI, including 18 ginsenosides and 10 aconite alkaloids. The HERB database (http://herb.ac.cn/), a TCM database guided by high-throughput experiments and references, was used to retrieve the number of herbal medicines containing the main active ingredients of SFI to observe the specificity of the active ingredients [26]. An ideal molecule that can act as a drug should comply with Lipinski's rule of ﬁve (RO5), which predicts the drug-likeness of the compound [27]. Therefore, we used the web tool SwissADME (http://www.swissadme.ch) to calculate the physicochemical properties of the compounds and predict the drug-likeness by RO5 [28]. Compounds that do not comply with RO5 were not considered to be active ingredients in SFI. Drug toxicology is an integral part of clinical research. The toxicities of the active ingredients of SFI were determined by the ProTox-II web server (https://tox-new.charite.de/protox_II) [29].

### 2.2. Collection of SFI Main Active Ingredients and Sepsis Targets

The PubChem database (https://pubchem.ncbi.nlm.nih.gov) was used to determine the canonical SMILES of the main active ingredients in SFI. Then, we input canonical SMILES into SwissTargetPrediction (http://www.swisstargetprediction.ch) [30], which can predict molecular targets, and species select “*Homo sapiens*,” to get the relevant targets of active ingredients. Among them, the screening standard of the targets were with probability>0.

GeneCards (https://www.genecards.org) is a comprehensive database that integrates various gene-centric data including genome, proteome, and transcriptome [31]. We used “sepsis” as the keyword to search the GeneCards database for related targets involved in sepsis. In addition, studies have shown that the degradation of glycocalyx is a key mechanism of action in sepsis [24], and our research group also found that SFI can inhibit the degradation of glycocalyx [13]. Therefore, we used “glycocalyx” as the keyword to search for related targets in the GeneCards database. Venny 2.1.0 (http://bioinfogp.cnb.csic.es/tools/venny) was used to find common targets of SFI-related targets and disease (glycocalyx and sepsis)-related targets, which are potential therapeutic targets of SFI involved in glycocalyx and sepsis pathways.

### 2.3. Functional Classification of Potential Therapeutic Targets of SFI Active Ingredients

Functional classification of potential therapeutic targets of SFI active ingredients was performed by the PANTHER classification system (http://www.pantherdb.org) which is a comprehensive system covering gene function classification, pathways, and statistical analysis [32]. We uploaded the therapeutic targets to the PANTHER classification system and selected the organism as “*Homo sapiens*” for functional classification to further analyze the mechanism of the targets on sepsis. The results were plotted by Microsoft Excel software (version 2019, Redmond, WA, United States).

### 2.4. PPI Network Construction

To identify the key targets related to sepsis pathophysiology, we put the obtained therapeutic targets for protein-protein interaction (PPI) analysis. The STRING 11.5 database (https://string-db.org) was used for PPI analysis and the results were visualized by Cytoscape 3.7.2. The organism was set to “*Homo sapiens*” and the minimum interaction score was set to 0.4. Afterward, we used the tool MCODE to cluster the PPI network and screen the core targets according to the degree value and the mcode node status.

In addition, the Gene Expression Omnibus (GEO) dataset was used for differential analysis of core target expression between sepsis patients and normal subjects to demonstrate the specificity of the core targets. We used “sepsis” as the keyword and “*Homo sapiens*” as the species to filter the eligible datasets. GraphPad Prism software (GraphPad Software Inc., La Jolla, CA, USA) was used for data analysis and visualization of the results. *P* value <0.05 was considered statistically significant.

### 2.5. Enrichment Analysis

The DAVID 6.8 (https://david.ncifcrf.gov/summary.jsp) [33] was used for enrichment analysis including GO biological function analysis and KEGG pathway analysis [33]. In the database settings, the identifier selected was “gene symbol”, and the species selected was “*Homo sapiens*”. The results with *P* value <0.05 were considered statistically significant and could be further analyzed.

### 2.6. Molecular Docking

Molecular docking could simulate the interaction of protein targets and small molecule compounds. AutoDock Vina_1.1.2 simulated the interaction of core targets with key active ingredients by using a semiflexible molecular docking model, and its docking results were evaluated by semiempirical free energy functions [34]. Specifically, the molecular structures of the key active ingredients of SFI in the mol2 format was obtained from the TCMSP database (https://tcmsp-e.com/). The molecular structure of the core targets in the PDB format was obtained from the RCSB PDB database (https://www.rcsb.org/), where the screening criteria for PDB crystal structures were determined according to the resolution of the protein and whether they have ligands. Therefore, we selected IL2 (PDB ID : 1M48) [35], BCHE (PDB ID : 6EQP) [36], AKT1 (PDB ID : 3OS5) [37], and LGALS3 (PDB ID : 6Q17) [38] in the RCSB PDB database for further processing. And, we used PyMol to complete the ligand separation of protein, the removal of water molecules, and the addition of hydrogen atoms. The selection of proteins and compounds as ligands and receptors was performed using the AutoDock tool. The docking of ligand molecules and proteins was performed by the blind docking method [39], and the whole protein receptor was covered with a grid box for binding site searching. A maximum of 10 conformations were considered for each compound, the exhaustiveness was set to 10, and the system default parameters were used for the rest of the settings. AutoDock Vina was used to dock the active ingredients and the targets, and obtained the aﬃnity of both. PyMOL 2.5.2 was used for visualization of the molecular docking results.

## 3. Results

### 3.1. Main Active Ingredients of SFI and Their Pharmacological Parameters

**Figure 2:**
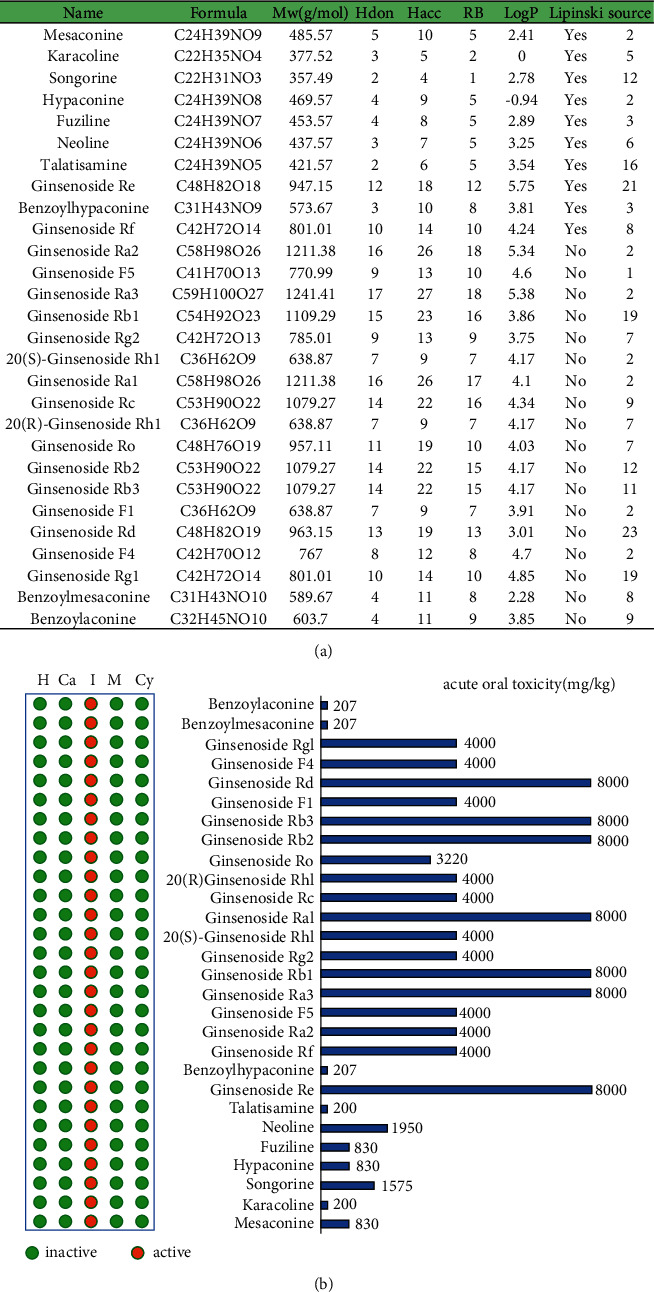
Main active ingredients of Shenfu injection (SFI) and their evaluation of pharmacological and toxicological parameters. (a) Physicochemical properties and distribution of active ingredients in SFI. (b) The toxicological parameters of the active ingredients in SFI. H: hepatotoxicity; Ca: carcinogenicity; I: immunotoxicity; M: mutagenicity; Cy: cytotoxicity.

Based on a recent UHPLC-QQQ MS study [25], a total of 28 active ingredients of SFI were obtained, including 18 ginsenosides and 10 aconite alkaloids. We used the HERB database to search for active ingredients in SFI to analyze their distribution in Chinese herbal medicines, and we can see that the distribution of active ingredients was specific. Among them, ginsenoside F5 only exists in 1 kind of Chinese herbal medicine, and mesaconine, hypaconine, ginsenoside Ra2, ginsenoside Ra3, 20 (S)-ginsenoside Rh1, ginsenoside Ra1, ginsenoside F1, and ginsenoside F4 only exist in 2 kinds of Chinese herbal medicines. The most widely distributed ginsenoside Rd is only found in 23 kinds of Chinese herbal medicines. SwissADME predicted the physicochemical properties of the main active ingredients of SFI and predicted drug-likeness by RO5 (Figure 2(a)). A total of 10 active ingredients are RO5 compliant, including 2 ginsenosides and 8 aconite alkaloids. The predictions of the ProTox-II web server showed the toxicological parameters of the active ingredients of SFI, including hepatotoxicity, carcinogenicity, immunotoxicity, mutagenicity, cytotoxicity, and acute oral toxicity (LD50, mg/kg). None of the active ingredients showed hepatotoxicity, carcinogenicity, mutagenicity, and cytotoxicity, while mesaconine, fuziline, neoline, ginsenoside Re, Rf, Ra1, Ra2, Ra3, Rb1, Rb2, Rb3, Rd, Rg2, Rc, Ro, Rg1, F1, F4, F5, 20 (S)-ginsenoside Rh1, 20 (R)-ginsenoside Rh1, benzoylmesaconine, and benzoylaconine have the risk of immune dysfunction. In addition, karacoline and talatisamine have the lowest LD50 values (200 mg/kg) (Figure 2(b)).

These analysis results showed that mesaconine, karacoline, songorine, hypaconine, fuziline, neoline, talatisamine, ginsenoside Re, benzoylhypaconine, and ginsenoside Rf complied with RO5, exhibited good pharmacological parameters, and could be used as active ingredients in SFI for further analysis.

### 3.2. Data Collection of SFI and Sepsis-Related Targets

**Figure 3:**
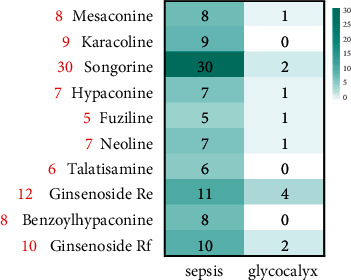
The heatmap shows targets of SFI active components associated with glycocalyx and sepsis pathways. The red numbers on the left represent the number of targets associated with glycocalyx and sepsis.

We input the canonical SMILES of the active ingredients of SFI into SwissTargetPrediction and got 129 targets. To explore the mechanism of action of the active ingredients in SFI in the treatment of sepsis, we screened the targets from a pathophysiological perspective. In the GeneCards database, we got 2777 targets involved in sepsis and 156 targets involved in glycocalyx. Afterward, we obtained 59 overlapping targets of SFI and diseases (sepsis and glycocalyx). The number of active ingredient targets associated with glycocalyx and sepsis is shown in Figure 3. Among them, songorine (30 targets, including 30 sepsis targets and 2 glycocalyx targets) is the most associated with the targets of pathophysiological processes, followed by ginsenoside Re (12 targets), ginsenoside Rf (10 targets), and karacoline (9 targets), indicating that these ingredients are most likely to be the key active ingredients of SFI in the treatment of sepsis.

### 3.3. Functional Classification of Potential Therapeutic Targets of SFI Active Ingredients

**Figure 4:**
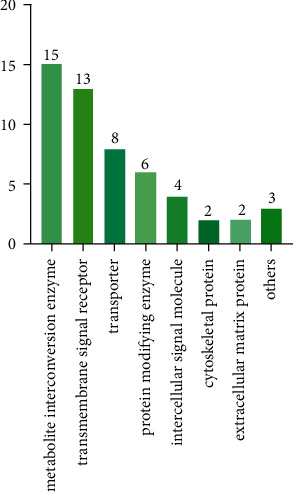
Panther classification: potential therapeutic targets of SFI for sepsis were classified. The numbers in the figure represent the number of targets for a given classification.

The PANTHER classification system was used for the functional classification of potential therapeutic targets. 53 out of 59 potential targets could be functionally classified. The 53 SFI targets related to sepsis and glycocalyx were divided into 8 different categories according to the protein class (Figure 4). Specifically, the metabolite interconversion enzyme (PC00262, 15 targets) was the most abundant category, followed by the transmembrane signal receptor (PC00197, 13 targets), and the transporter (PC00227, 8 targets). Among metabolite interconversion enzymes, 5 targets are glycosidases, which are closely related to the conversion of the glycocalyx.

### 3.4. PPI Network Construction

**Figure 5:**
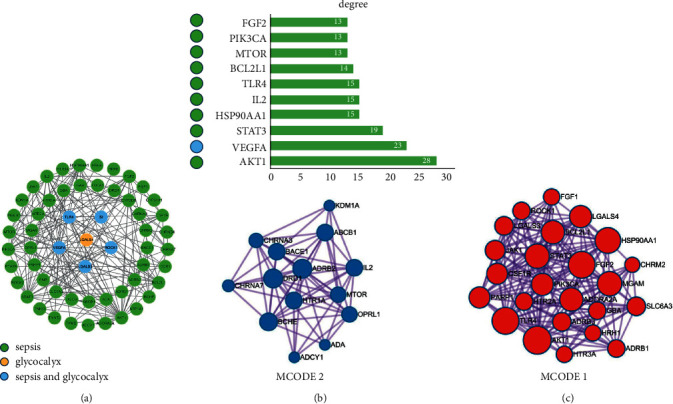
PPI network. (a) The PPI network was constructed for the 59 potential therapeutic targets of SFI associated with glycocalyx and sepsis. Nodes in green, orange, and blue represent SFI targets correlated with “sepsis,” “glycocalyx,” and “sepsis and glycocalyx.” (b) The top ten targets in the PPI network by the degree value. The color of the node on the left side of the targets was related to “sepsis,” “glycocalyx,” and “sepsis and glycocalyx,” respectively. (c) Two clusters of MCODE cluster analysis.

The obtained overlapping targets were entered into the STRING 11.5 database for PPI and visualized by Cytoscape 3.7.2. Not all targets in the sepsis pathway are related to glycocalyx, and there are 6 targets (SI, ROCK1, TLR4, VEGFA, HPSE, and LGALS3) of active ingredients not only related to glycocalyx, but also involved in the sepsis pathway. So, 59 therapeutic targets were constructed as a PPI network with 56 nodes, 191 edges, and an average degree of 5 (Figure 5(a)). And, as shown in Figure 5(b), the top ten targets ranked by the degree value are AKT1, VEGFA, STAT3, HSP90AA1, IL2, TLR4, BCL2L1, MTOR, PIK3CA, and FGF2.

Through the clustering module analysis of MCODE, we obtained 2 gene clusters as shown in Figure 5(c). The seed of the mcode node status in each gene cluster is LGALS3 and BCHE, and the highest degree value in each gene cluster is AKT1 and IL2. Therefore, LGALS3, BCHE, AKT1, and IL2 are the core targets. Among them, BCHE, AKT1, and IL2 are involved in the sepsis pathway, and LGALS3 is involved in both the sepsis and glycocalyx pathways.

### 3.5. Differential Expression Analysis

**Figure 6:**
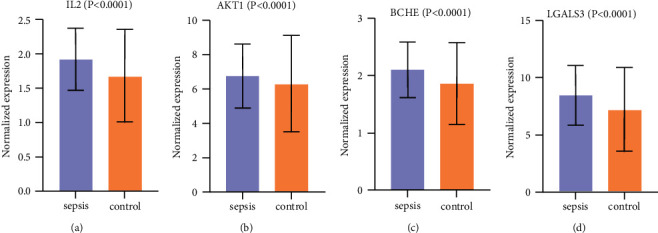
Differential expression analysis of the core targets in the chip datasets GSE134347 and GSE6535.

The chip datasets GSE134347 and GSE6535 from the GEO database were used to validate the differential expression of the core targets. Among them, the GSE134347 dataset contains 156 sepsis samples and 83 normal samples, and GSE6535 contains 12 sepsis samples and 17 normal samples. GraphPad Prism software was used for differential analysis and visualization of the results (Figure 6). Validated by the GEO datasets, the core targets are the differentially expressed genes of sepsis. Specifically, AKT1, IL2, LGALS3, and BCHE are the upregulated targets.

### 3.6. Enrichment Analysis

To further analyze the synergistic mechanism of SFI in the treatment of sepsis, we used the DAVID 6.8 to analyze 59 active ingredient targets involved in sepsis and glycocalyx by GO biological function analysis and KEGG pathway analysis. Statistical differences with a *P* value < 0.05 in the enrichment analysis results could be further analyzed.

**Figure 7:**
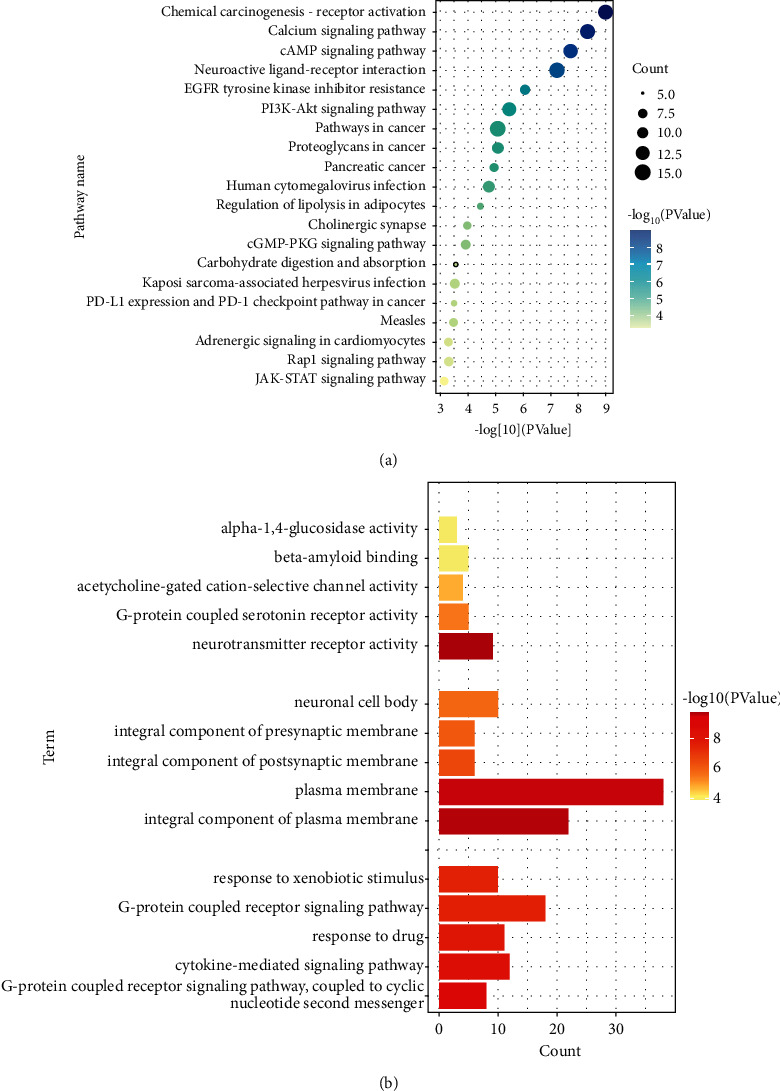
Enrichment analysis is as follows: (a) top 20 pathways of KEGG enrichment analysis; (b) top 5 functions of MF, CC, and BP in GO enrichment analysis.

KEGG enrichment analysis results showed that 91 of the 110 pathways have a *P* value <0.05. We showed the top 20 pathways based on the *P* value (Figure 7(a)). Among them, the calcium signaling pathway, cAMP signaling pathway, PI3K−Akt signaling pathway, Rap1 signaling pathway, and Jak−STAT signaling pathway may be the potential therapeutic mechanisms of SFI for sepsis.

GO analysis was screened according to a *P* value <0.05, showing that there are 185 items of biological process (BP), 31 items of cellular component (CC), and 39 items of molecular function (MF). We demonstrated each of the top five functions of BP, CC, and MF (Figure 7(b)). GO analysis showed that the MF of SFI was mainly related to the activities of glucosidase, G-protein, and neurotransmitter receptor, and beta-amyloid binding. The CC was mainly associated with the plasma membrane, synapses, and neuronal cell bodies. And, the BP was mainly related to the G-protein coupled receptor signaling pathway, the response to heterologous stimuli and drugs, and the cytokine-mediated signaling pathway.

### 3.7. Molecular Docking

We docked the core targets IL2 (PDB ID : 1M48), BCHE (PDB ID : 6EQP), AKT1 (PDB ID : 3OS5), and LGALS3 (PDB ID : 6Q17) with the key active ingredients; the lower the binding energy, the better the combination of the two. Studies have shown that affinity lower than −4.25 kcal/mol hasa certain binding force, the affinity lower than −5.0 kcal/mol has a good binding force, and the affinity lower than −7.0 kcal/mol has a strong binding force [40, 41].The results showed that all key active ingredients had a good binding ability to the core targets (Table 1). In addition, we docked the standard molecule of the protein with the protein, and the results also showed that the SFI active components had stronger binding ability to the core protein targets compared with the standard molecules (Supplementary Table S1).

**Figure 8:**
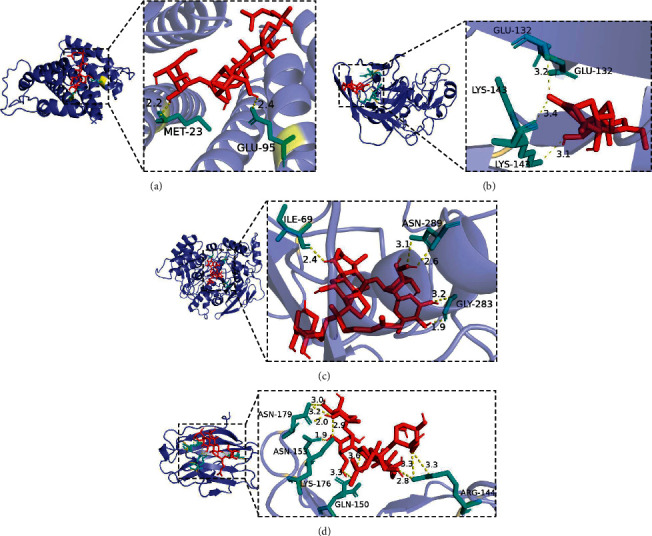
Molecular docking: (a) ginsenoside Rf and IL2, affinity = −7.9 kcal/mol; (b) ginsenoside Re and BCHE, affinity = −10.6 kcal/mol; (c) songorine and AKT1, affinity = −8.0 kcal/mol; (d) ginsenoside Re and LGALS3, affinity = −7.2 kcal/mol.

**Figure 9:**
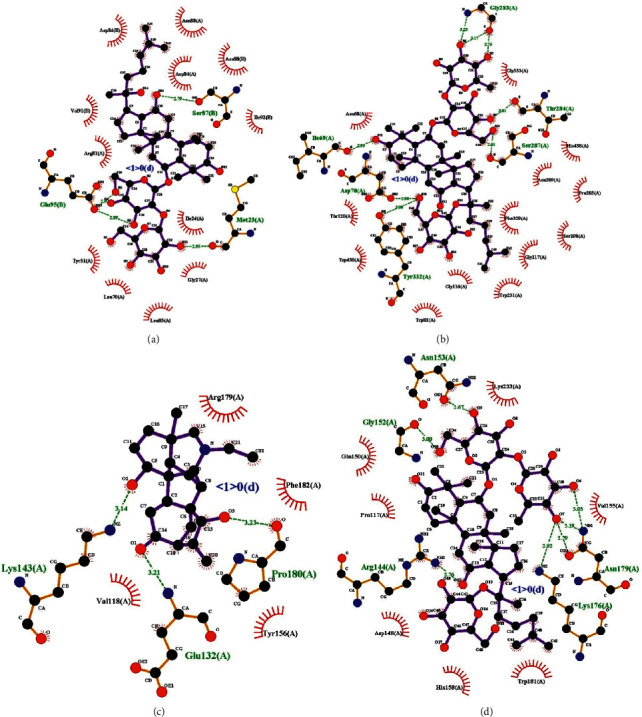
Interactions between compounds and amino acids in protein binding sites; (a) ginsenoside Rf and IL2; (b) ginsenoside Re and BCHE; (c) songorine and AKT1; (d) ginsenoside Re and LGALS3.

We presented targets with the strongest affinity (Figure 8), and Table 2 shows the bond length of the interacting residues. Studies indicated that the hydrogen bond distance in the range of 2.8–3.2 Å is favorable for bonding [42]. The median distance of the hydrogen bond between amide C=O and OH is 2.75 Å, while in the NH donors, the median distance increases to 2.9 Å. Our molecular docking results showed that there were some interacting residues in the protein targets that were beneficial to the binding of molecules to proteins, and the remaining interacting residues may be affected by rigid docking, which has the possibility of further optimization and research. Furthermore, we present the interactions of all interacting residues with the protein-ligand complexes in Figure 9, which further demonstrates that key ingredients of SFI have good binding capacity to core targets.

## 4. Discussion

Sepsis remains the most important cause of death in global healthcare despite numerous clinical and experimental studies. Sepsis involves not only inflammatory responses to infection but also nonimmune pathways which include cardiovascular, coagulation, and metabolism. At present, studies have found that glycocalyx can regulate leukocyte adhesion and the homeostasis of the vascular system. Sepsis promotes glycocalyx degradation, which could lead to microvascular thrombosis and enhanced leukocyte adhesion. The level of glycocalyx was correlated with the degree of organ dysfunction and mortality [24]. Therefore, this study adopted a new strategy to explore the therapeutic mechanism and key active ingredients of SFI by combining the sepsis pathway and glycocalyx from the perspective of sepsis pathophysiology. This study identified 28 active constituents of SFI, including 18 ginsenosides and 10 aconite alkaloids. Among them, songorine, ginsenoside Rf, ginsenoside Re, and karacoline were the key active components of SFI in the treatment of sepsis. There were 59 targets in SFI related to glycocalyx and sepsis pathways. IL2, AKT1, BCHE, and LGALS3 were considered to be the core targets of SFI. Our study suggests that the potential therapeutic mechanism of SFI for sepsis may be related to the calcium signaling pathway, cAMP signaling pathway, PI3K−Akt signaling pathway, Rap1 signaling pathway, and Jak−STAT signaling pathway. We thought that the treatment of SFI for sepsis is based on anti-inflammatory, antioxidant, immune regulation, protection of endothelial cells, and improvement of the glycocalyx.

cAMP is a common member of the second messenger family that regulates key physiological processes including metabolism and calcium homeostasis. Elevation of intracellular cAMP inhibits the production of inflammatory mediators such as TNF-*α* and IL-12 [43]. Studies have shown that activation of the cAMP/PKA pathway alleviates the excessive inflammatory response in sepsis [44] and its induced cardiac insufficiency [45]. Calcium signaling and cAMP signaling interact at many levels and they regulate each other [46]. Among them, the cAMP/PKA pathway forms calcium signals in various cell types including cardiomyocytes and neurons [47, 48]. Calcium ion channels are involved in the occurrence and development of sepsis [49]; calcium overload is the main mechanism of systemic inflammatory response and endothelial cell injury in sepsis [50]. The accumulation of Ca2+ is an important cause of the progression from sepsis to multiple organ dysfunction [51]. Moreover, Rap1 performs intracellular and extracellular signal coupling through cAMP and Ca2+. Rap1 signaling interacts with the Ca2+ signaling pathway and has important cellular functions such as controlling platelet activation. Rap1 could act as a Ca2+ effector, but it could also regulate the level of Ca2+ [52]. And, sepsis significantly downregulates Rap1 expression, resulting in increased vascular permeability and endothelial cell dysfunction [53].

The PI3K-Akt signaling pathway regulates multiple essential cellular functions such as transcription, growth, and multiplication. Activation of the PI3K−Akt pathway inhibits inflammation [54], fights apoptosis, improves oxidative responses in sepsis [55], and attenuates sepsis-induced myocardial injury [56]. Similar to PI3K, Jak-STAT is a canonical inflammatory pathway. It is not only involved in the regulation of the immune response of sepsis [57], but also related to the dysfunction of multiple organs and plays a key role in the treatment of sepsis [58].

According to the analysis of ingredients and targets, we determined that songorine, ginsenoside Rf, ginsenoside Re, and karacoline were the key active ingredients of SFI. Among them, songorine and karacoline belong to aconite alkaloids, and ginsenoside Rf and ginsenoside Re belong to ginsenosides. Studies have shown that *Aconitum carmichaeli Debx* and its active ingredients have various effects including anti-inflammatory and analgesic, immune system, and energy metabolism [59]. At present, studies have confirmed that songorine helps to enhance the oxidative defense of septic mice, promote myocardial mitochondrial synthesis, and improve myocardial injury in sepsis [60]. Ginsenosides inhibit the activation of inflammasomes [61] and improve acute lung injury and acute respiratory distress syndrome in sepsis [62]. Ginsenosides Rg1 and Re enhance immune response, inhibit the production and expression of various proinflammatory mediators stimulated by LPS, and improve the survival rate of sepsis [63]. Ginsenosides Re significantly attenuated LPS-induced inflammatory response and cardiac insufficiency [64].

Among the functional classifications of therapeutic targets for SFI, the metabolite interconversion enzyme was the most abundant class. Among them, 8 targets are hydrolase, 4 targets are transferase, 3 targets are oxidoreductase, and 1 target is lyase. It can be seen that SFI participates in many key biological processes in the human body. In addition, in the metabolite interconversion enzyme, 5 targets are glycosidases, which are closely related to the conversion of the glycocalyx. Research supports that various glycosidases, including heparanase and hyaluronidase, are involved in the degradation of glycocalyx and play an important role in the pathophysiological mechanism of sepsis [24, 65–67].

According to the cluster analysis of MCODE and the validation on the GEO dataset, we obtained four core targets of LGALS3, BCHE, AKT1, and IL2. LGALS3 is a carbohydrate-binding protein that affects angiogenesis by affecting VEGFR2 [68]. Interaction of multicarbon compounds bound with LGALS3 with transmembrane mucins maintains the integrity of glycocalyx. LGALS3 has an important role in the barrier function of glycocalyx [69].

BCHE plays an important role in the inflammatory response [70] and could be used as a marker of systemic inflammatory response. Studies suggested that BCHE may be an important therapeutic target for sepsis and has a significant predictive power for mortality in critically ill patients [71].

AKT1 is critical for acute inflammation, mainly by regulating neutrophils and monocytes [72]. In sepsis, downregulation of AKT1 regulates inflammatory responses and plays a role in immunosuppression [73, 74]. IL2 also plays an important role in immune homeostasis and inflammatory responses [75, 76]. In sepsis, IL2 is also an important marker and diagnostic reference [77].

The molecular docking results of the core targets and the key active ingredients of SFI show that the two have a good binding ability, which further proves that the treatment of SFI for sepsis is based on anti-inflammatory, antioxidant, immune regulation, protection of endothelial cells, and improvement of the glycocalyx.

This study adopted a new research strategy to explore and identify the key active ingredients of SFI from the perspective of the pathophysiology of sepsis, improving the accuracy of potential therapeutic targets for SFI in the treatment of sepsis. And we obtained the blood-introducing active ingredients of SFI according to the UHPLC-QQQ MS study, avoiding the use of public databases to screen out a large number of nonspecific ingredients.

However, this study also has some shortcomings. First, based on the limitations of network pharmacology, we could not consider the effect of the SFI dose on disease. Second, the present network analysis is static, which contradicts the dynamic changes in the occurrence and development of the disease. Finally, although animal experiments and clinical studies have shown that SFI has an obvious therapeutic effect on sepsis, the research on the active ingredients of SFI is still insufficient, and further research on its adverse effects is required.

## 5. Conclusions

Based on network pharmacology, this study explored the mechanism of SFI in the treatment of sepsis from the perspective of pathophysiology. Songorine, ginsenoside Rf, ginsenoside Re, and karacoline were identified as key active ingredients in SFI for the treatment of sepsis. LGALS3, BCHE, AKT1, and IL2 were identified as core targets for the potential treatment of SFI. We provide a new perspective for the study of TCM network pharmacology and candidate compounds for drug development. However, its clinical application still needs further research and confirmation.

## Figures and Tables

**Table 1:** Molecular docking results.

Compound	Structure	Binding energy (kcal/mol)			
		IL2	BCHE	AKT1	LGALS3
Songorine	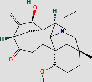	−7.5/−7.5/−7.2/−7.2/−7.2	−8.7/−8.6/−8.6/−8.5/−8.5	−8.0/−8.0/−7.7/−7.7−7.5	−6.6/−6.6/−6.6/−6.5/−6.4

Ginsenoside Rf	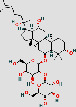	−7.9/−7.4/−7.4/−7.3/−7.2	−9.0/−8.9−8.6/−8.4/−8.3	−7.7/−7.6/−7.2/−7.1/−7.1	−6.4/−6.4/−6.3/−6.2/−6.2

Ginsenoside Re	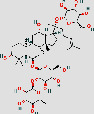	−7.5/−7.5/−7.4/−7.1/−7.1	−10.6/−10.4/−10.0/−9.6/−9.5	−7.6/−7.5/−7.2/−7.2/−7.2	−7.2/−7.2/−7.1/−7.1/−7.1

Karacoline	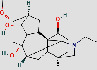	−7.2/−7.1/−7.0/−6.9/−6.7	−8.6/−8.2/−8.1/−8.0/−8.0	−7.3/−7.3/−7.3/−7.2/−7.2	−6.3/−6.2/−6.0/−6.0/−5.9

The figures in the table are the top 5 binding energy of molecular docking analysis.

**Table 2:** The bond length of the interacting residues.

	H1	H2	H3	H4	H5
IL2	MET−23 (2.2)	GLU−95 (2.4)			
BCHE	GLU−283 (1.9/3.2)	ASN−289 (2.6/3.1)	ILE−69 (2.4)		
AKT1	ILE−69 (2.4)	GLY−283 (3.2/1.9)	ASN−289 (2.6/3.1)		
LGALS3	ARG−144 (2.8/3.3/3.3)	GLN−150 (3.3/3.6)	ASN−153 (1.9)	LYS−176 (2.9)	ASN−179 (2.0/3.0/3.2)

H: hbond interaction.

## Data Availability

The data used to support the findings of this study are included within the article and supplementary materials.

## Supplementary Materials

Supplementary Materials Supplementary Table S1: Binding energies of protein targets docked with SFI active ingredients and standard molecules.
