# Supplementary material for: Therapeutic Mechanism and Key Active Ingredients of Shenfu Injection in Sepsis: A Network Pharmacology and Molecular Docking Approach
Source: Evid Based Complement Alternat Med. 2022 Aug 26;2022:9686149. doi: 10.1155/2022/9686149 (PMC9439916; doi:10.1155/2022/9686149)
Supplement: Supplementary Materials — Supplementary Table S1: Binding energies of protein targets docked with SFI active ingredients and standard molecules. [file 9686149.f1.pdf]

Supplementary Table S1 | Binding energies of protein targets docked with SFI active ingredients and standard molecules.

Compound	Binding energy/ (kcal/mol)			
	IL-2	BCHE	AKT1	LGALS3
Songorine	-7.5	-8.9	-8.0	-6.6
Ginsenoside Rf	-7.9	-9.0	-7.7	-6.4
Ginsenoside Re	-7.5	-10.6	-7.6	-7.2
Karacoline	-7.2	-8.6	-7.3	-6.3
Standard molecules	-6.5	-6.7	-7.6	-6.6
